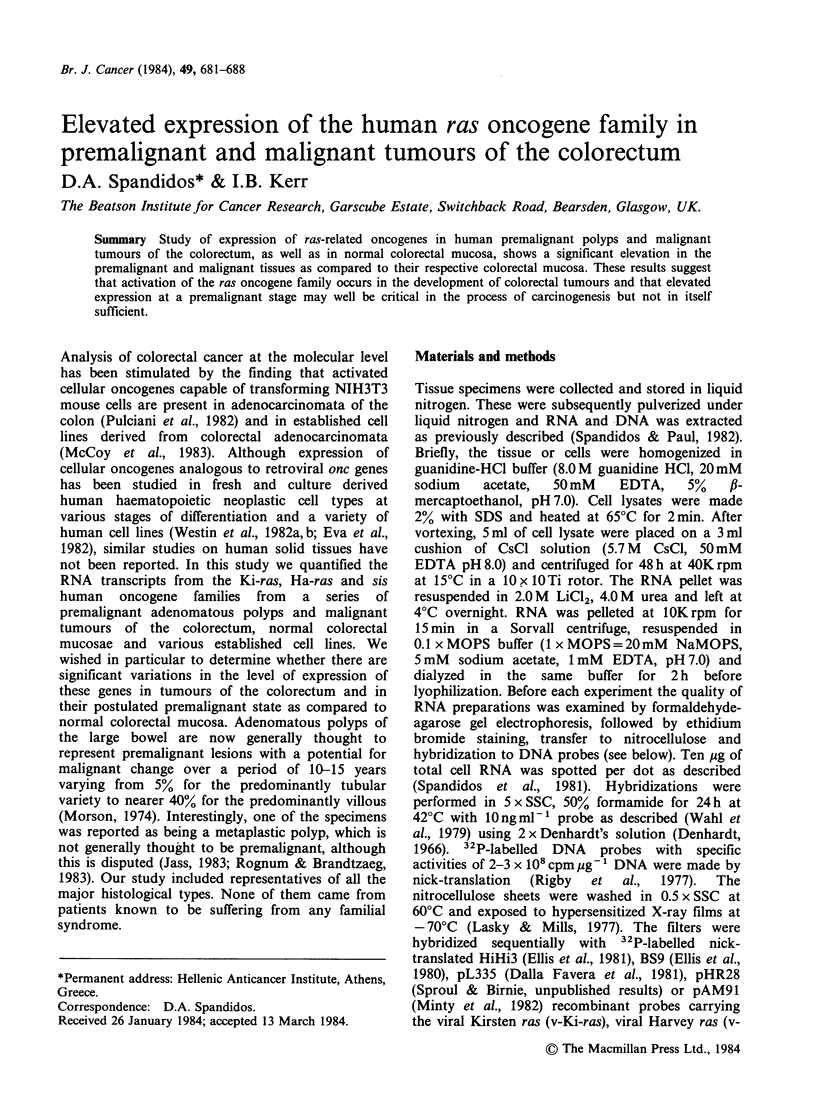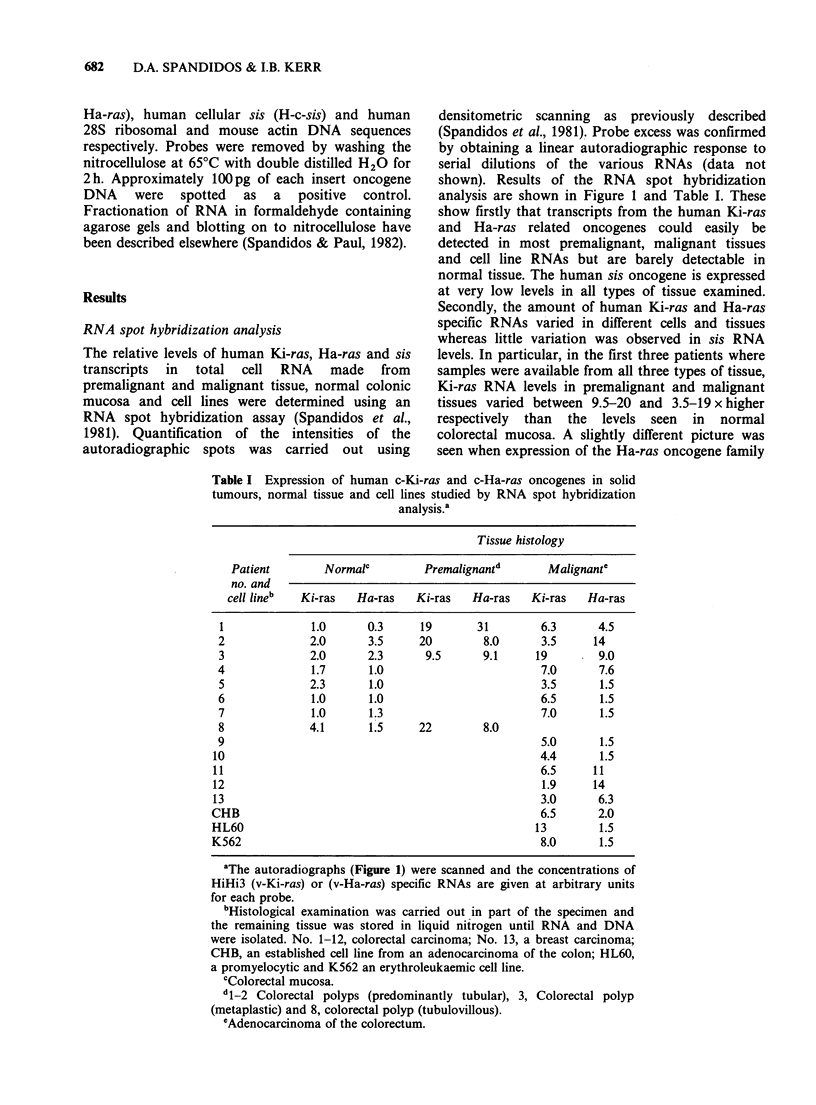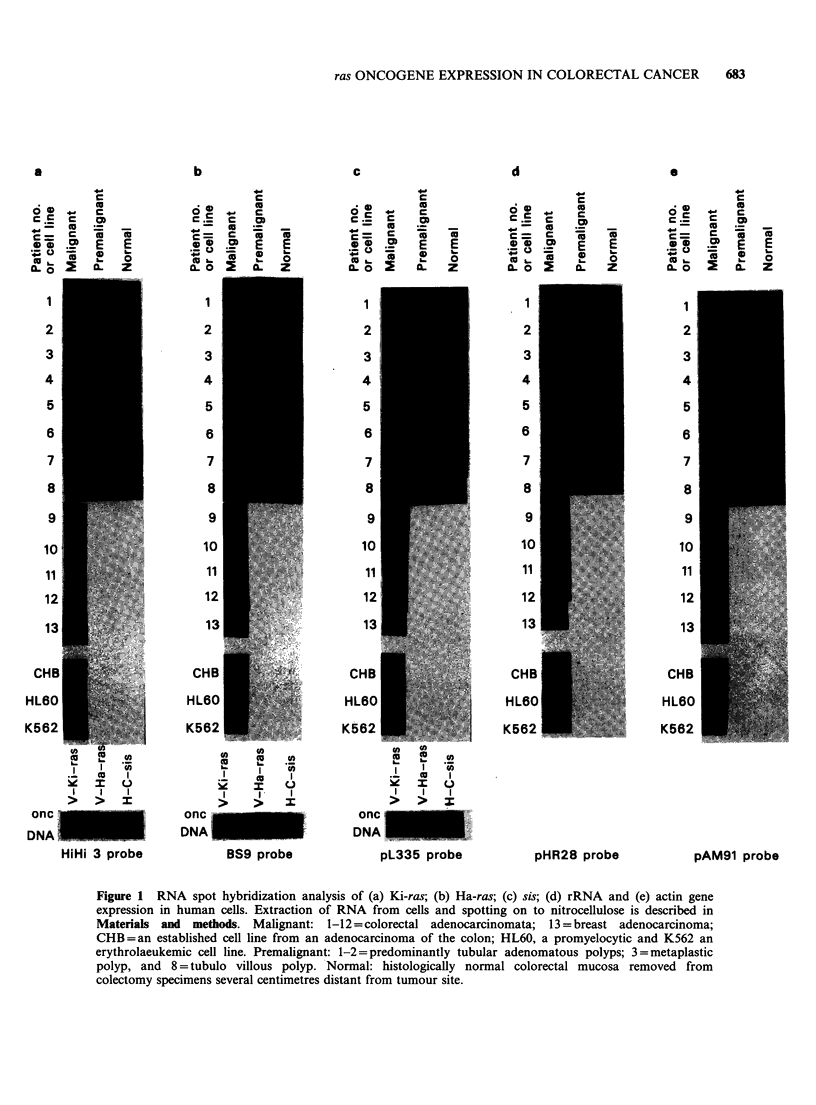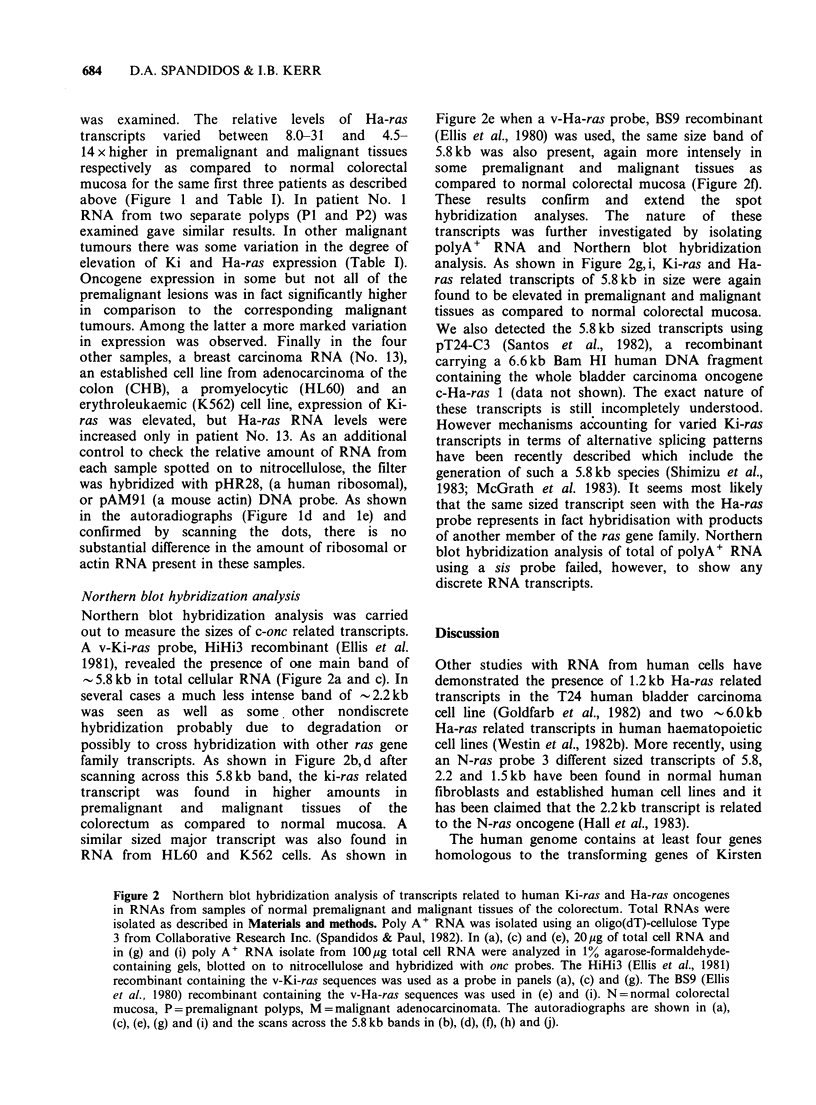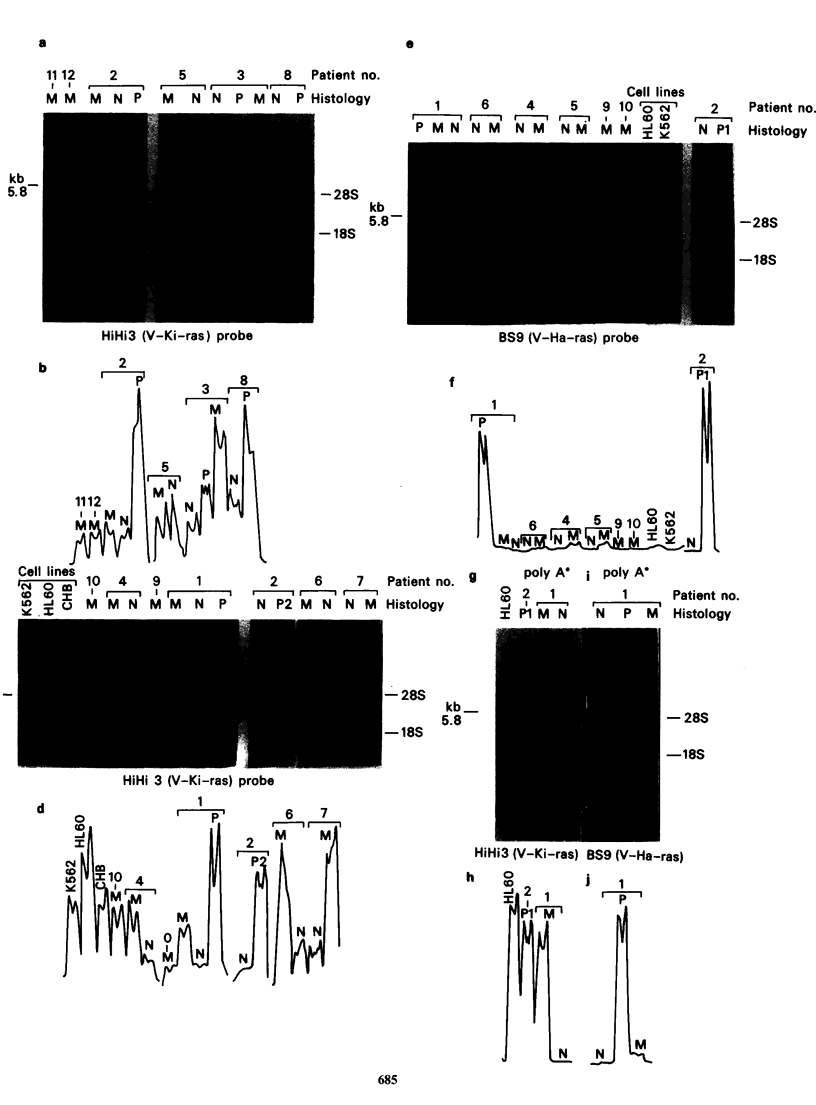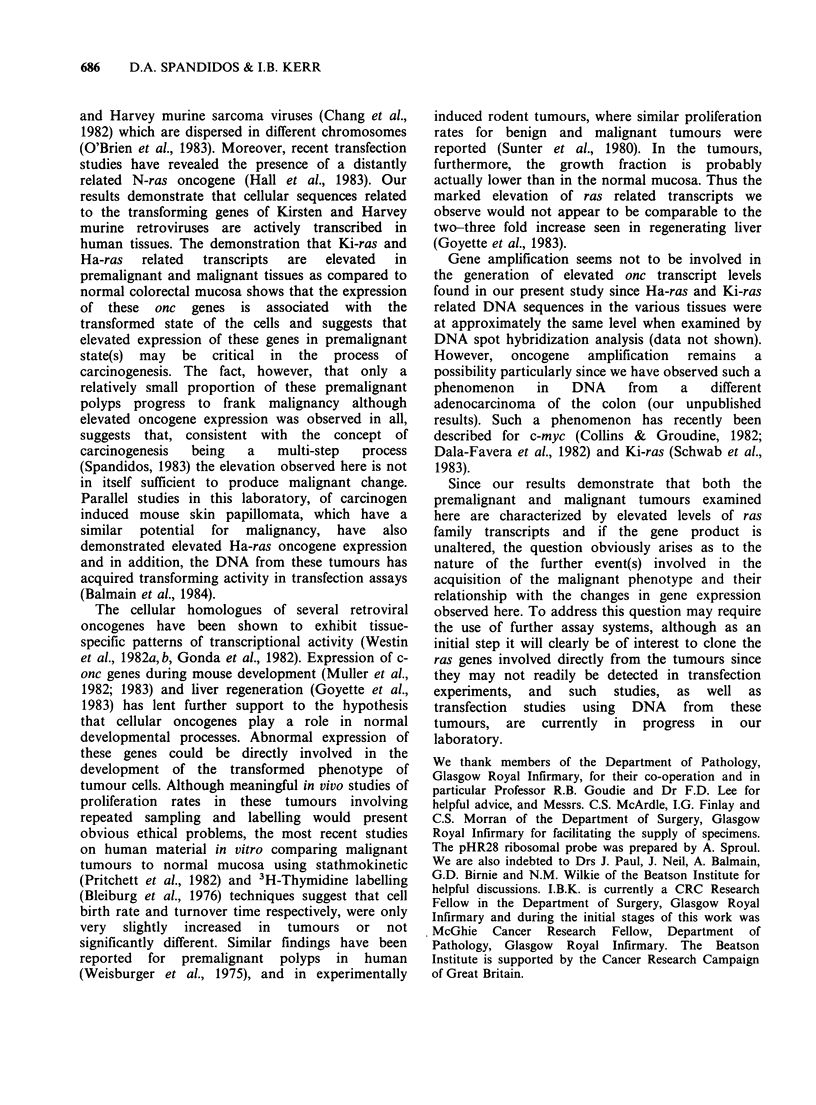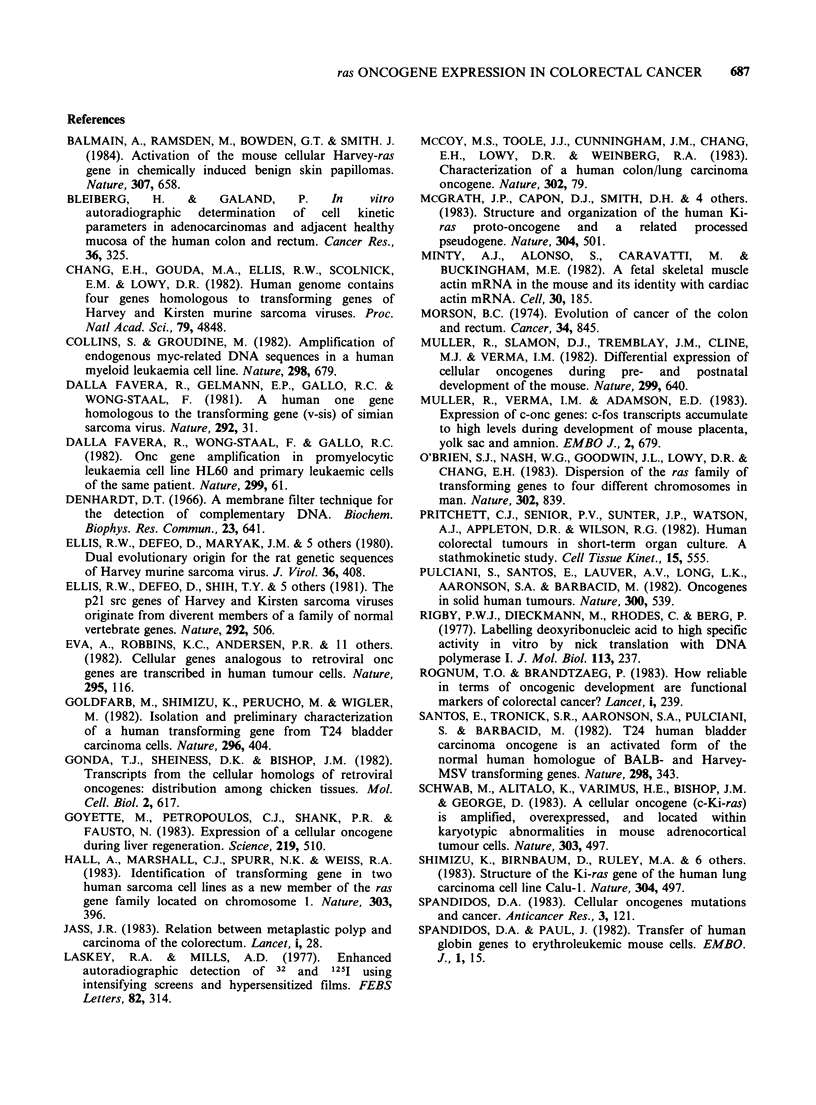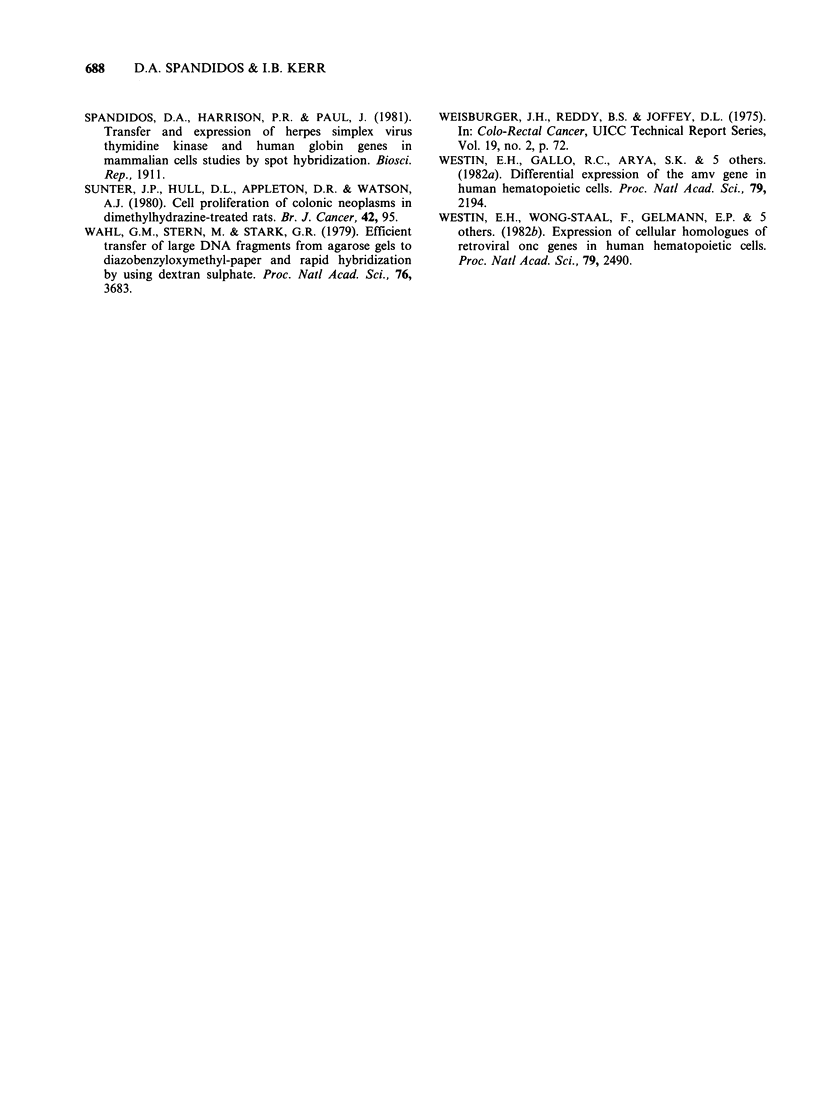# Elevated expression of the human ras oncogene family in premalignant and malignant tumours of the colorectum.

**DOI:** 10.1038/bjc.1984.108

**Published:** 1984-06

**Authors:** D. A. Spandidos, I. B. Kerr

## Abstract

**Images:**


*Br. J. Cancer* (1984), **49**, 681–688

### Elevated expression of the human *ras* oncogene family in premalignant and malignant tumours of the colorectum

D.A. Spandidos* & I.B. Kerr

*The Beatson Institute for Cancer Research, Garscube Estate, Switchback Road, Bearsden, Glasgow, UK.*

**Summary** Study of expression of *ras*-related oncogenes in human premalignant polyps and malignant tumours of the colorectum, as well as in normal colorectal mucosa, shows a significant elevation in the premalignant and malignant tissues as compared to their respective colorectal mucosa. These results suggest that activation of the *ras* oncogene family occurs in the development of colorectal tumours and that elevated expression at a premalignant stage may well be critical in the process of carcinogenesis but not in itself sufficient.

Analysis of colorectal cancer at the molecular level has been stimulated by the finding that activated cellular oncogenes capable of transforming NIH3T3 mouse cells are present in adenocarcinomata of the colon (Pulciani *et al.*, 1982) and in established cell lines derived from colorectal adenocarcinomata (McCoy *et al.*, 1983). Although expression of cellular oncogenes analogous to retroviral *onc* genes has been studied in fresh and culture derived human haematopoietic neoplastic cell types at various stages of differentiation and a variety of human cell lines (Westin *et al.*, 1982a, b; Eva *et al.*, 1982), similar studies on human solid tissues have not been reported. In this study we quantified the RNA transcripts from the Ki-*ras*, Ha-*ras* and *sis* human oncogene families from a series of premalignant adenomatous polyps and malignant tumours of the colorectum, normal colorectal mucosae and various established cell lines. We wished in particular to determine whether there are significant variations in the level of expression of these genes in tumours of the colorectum and in their postulated premalignant state as compared to normal colorectal mucosa. Adenomatous polyps of the large bowel are now generally thought to represent premalignant lesions with a potential for malignant change over a period of 10–15 years varying from 5% for the predominantly tubular variety to nearer 40% for the predominantly villous (Morson, 1974). Interestingly, one of the specimens was reported as being a metaplastic polyp, which is not generally thought to be premalignant, although this is disputed (Jass, 1983; Rognum & Brandtzaeg, 1983). Our study included representatives of all the major histological types. None of them came from patients known to be suffering from any familial syndrome.

*Permanent address: Hellenic Anticancer Institute, Athens, Greece.

Correspondence: D.A. Spandidos.

Received 26 January 1984; accepted 13 March 1984.

### Materials and methods

Tissue specimens were collected and stored in liquid nitrogen. These were subsequently pulverized under liquid nitrogen and RNA and DNA was extracted as previously described (Spandidos & Paul, 1982). Briefly, the tissue or cells were homogenized in guanidine-HCl buffer (8.0 M guanidine HCl, 20 mM sodium acetate, 50 mM EDTA, 5% $\beta$-mercaptoethanol, pH 7.0). Cell lysates were made 2% with SDS and heated at 65°C for 2 min. After vortexing, 5 ml of cell lysate were placed on a 3 ml cushion of CsCl solution (5.7 M CsCl, 50 mM EDTA pH 8.0) and centrifuged for 48 h at 40K rpm at 15°C in a 10 x 10Ti rotor. The RNA pellet was resuspended in 2.0 M $LiCl_2$, 4.0 M urea and left at 4°C overnight. RNA was pelleted at 10K rpm for 15 min in a Sorvall centrifuge, resuspended in 0.1 x MOPS buffer (1 x MOPS = 20 mM NaMOPS, 5 mM sodium acetate, 1 mM EDTA, pH 7.0) and dialyzed in the same buffer for 2 h before lyophilization. Before each experiment the quality of RNA preparations was examined by formaldehyde-agarose gel electrophoresis, followed by ethidium bromide staining, transfer to nitrocellulose and hybridization to DNA probes (see below). Ten $\mu$g of total cell RNA was spotted per dot as described (Spandidos *et al.*, 1981). Hybridizations were performed in 5 x SSC, 50% formamide for 24 h at 42°C with 10 ng $ml^{-1}$ probe as described (Wahl *et al.*, 1979) using 2 x Denhardt's solution (Denhardt, 1966). $^{32}P$-labelled DNA probes with specific activities of $2–3 \times 10^8$ cpm $\mu g^{-1}$ DNA were made by nick-translation (Rigby *et al.*, 1977). The nitrocellulose sheets were washed in 0.5 x SSC at 60°C and exposed to hypersensitized X-ray films at −70°C (Lasky & Mills, 1977). The filters were hybridized sequentially with $^{32}P$-labelled nick-translated HiHi3 (Ellis *et al.*, 1981), BS9 (Ellis *et al.*, 1980), pL335 (Dalla Favera *et al.*, 1981), pHR28 (Sproul & Birnie, unpublished results) or pAM91 (Minty *et al.*, 1982) recombinant probes carrying the viral Kirsten *ras* (v-Ki-*ras*), viral Harvey *ras* (v-

© The Macmillan Press Ltd., 1984

Ha-*ras*), human cellular *sis* (H-c-*sis*) and human 28S ribosomal and mouse actin DNA sequences respectively. Probes were removed by washing the nitrocellulose at 65°C with double distilled $H_2O$ for 2 h. Approximately 100 pg of each insert oncogene DNA were spotted as a positive control. Fractionation of RNA in formaldehyde containing agarose gels and blotting on to nitrocellulose have been described elsewhere (Spandidos & Paul, 1982).

### Results

### *RNA spot hybridization analysis*

The relative levels of human Ki-*ras*, Ha-*ras* and *sis* transcripts in total cell RNA made from premalignant and malignant tissue, normal colonic mucosa and cell lines were determined using an RNA spot hybridization assay (Spandidos *et al*., 1981). Quantification of the intensities of the autoradiographic spots was carried out using densitometric scanning as previously described (Spandidos *et al*., 1981). Probe excess was confirmed by obtaining a linear autoradiographic response to serial dilutions of the various RNAs (data not shown). Results of the RNA spot hybridization analysis are shown in Figure 1 and Table I. These show firstly that transcripts from the human Ki-*ras* and Ha-*ras* related oncogenes could easily be detected in most premalignant, malignant tissues and cell line RNAs but are barely detectable in normal tissue. The human *sis* oncogene is expressed at very low levels in all types of tissue examined. Secondly, the amount of human Ki-*ras* and Ha-*ras* specific RNAs varied in different cells and tissues whereas little variation was observed in *sis* RNA levels. In particular, in the first three patients where samples were available from all three types of tissue, Ki-*ras* RNA levels in premalignant and malignant tissues varied between 9.5–20 and 3.5–19 × higher respectively than the levels seen in normal colorectal mucosa. A slightly different picture was seen when expression of the Ha-*ras* oncogene family

**Table I** Expression of human c-Ki-*ras* and c-Ha-*ras* oncogenes in solid tumours, normal tissue and cell lines studied by RNA spot hybridization analysis.[a]

| *Patient no. and cell line*[b] | *Tissue histology* | | | | | |
|---|---|---|---|---|---|---|
| | *Normal*[c] | | *Premalignant*[d] | | *Malignant*[e] | |
| | *Ki*-ras | *Ha*-ras | *Ki*-ras | *Ha*-ras | *Ki*-ras | *Ha*-ras |
| 1 | 1.0 | 0.3 | 19 | 31 | 6.3 | 4.5 |
| 2 | 2.0 | 3.5 | 20 | 8.0 | 3.5 | 14 |
| 3 | 2.0 | 2.3 | 9.5 | 9.1 | 19 | 9.0 |
| 4 | 1.7 | 1.0 | | | 7.0 | 7.6 |
| 5 | 2.3 | 1.0 | | | 3.5 | 1.5 |
| 6 | 1.0 | 1.0 | | | 6.5 | 1.5 |
| 7 | 1.0 | 1.3 | | | 7.0 | 1.5 |
| 8 | 4.1 | 1.5 | 22 | 8.0 | | |
| 9 | | | | | 5.0 | 1.5 |
| 10 | | | | | 4.4 | 1.5 |
| 11 | | | | | 6.5 | 11 |
| 12 | | | | | 1.9 | 14 |
| 13 | | | | | 3.0 | 6.3 |
| CHB | | | | | 6.5 | 2.0 |
| HL60 | | | | | 13 | 1.5 |
| K562 | | | | | 8.0 | 1.5 |

[a]The autoradiographs (**Figure 1**) were scanned and the concentrations of HiHi3 (v-Ki-*ras*) or (v-Ha-*ras*) specific RNAs are given at arbitrary units for each probe.

[b]Histological examination was carried out in part of the specimen and the remaining tissue was stored in liquid nitrogen until RNA and DNA were isolated. No. 1–12, colorectal carcinoma; No. 13, a breast carcinoma; CHB, an established cell line from an adenocarcinoma of the colon; HL60, a promyelocytic and K562 an erythroleukaemic cell line.

[c]Colorectal mucosa.

[d]1–2 Colorectal polyps (predominantly tubular), 3, Colorectal polyp (metaplastic) and 8, colorectal polyp (tubulovillous).

[e]Adenocarcinoma of the colorectum.

**Figure 1** RNA spot hybridization analysis of (a) Ki-*ras*; (b) Ha-*ras*; (c) *sis*; (d) rRNA and (e) actin gene expression in human cells. Extraction of RNA from cells and spotting on to nitrocellulose is described in **Materials and methods**. Malignant: 1–12 = colorectal adenocarcinomata; 13 = breast adenocarcinoma; CHB = an established cell line from an adenocarcinoma of the colon; HL60, a promyelocytic and K562 an erythrolaeukemic cell line. Premalignant: 1–2 = predominantly tubular adenomatous polyps; 3 = metaplastic polyp, and 8 = tubulo villous polyp. Normal: histologically normal colorectal mucosa removed from colectomy specimens several centimetres distant from tumour site.

was examined. The relative levels of Ha-*ras* transcripts varied between 8.0–31 and 4.5–14 × higher in premalignant and malignant tissues respectively as compared to normal colorectal mucosa for the same first three patients as described above (Figure 1 and Table I). In patient No. 1 RNA from two separate polyps (P1 and P2) was examined gave similar results. In other malignant tumours there was some variation in the degree of elevation of Ki and Ha-*ras* expression (Table I). Oncogene expression in some but not all of the premalignant lesions was in fact significantly higher in comparison to the corresponding malignant tumours. Among the latter a more marked variation in expression was observed. Finally in the four other samples, a breast carcinoma RNA (No. 13), an established cell line from adenocarcinoma of the colon (CHB), a promyelocytic (HL60) and an erythroleukaemic (K562) cell line, expression of Ki-*ras* was elevated, but Ha-*ras* RNA levels were increased only in patient No. 13. As an additional control to check the relative amount of RNA from each sample spotted on to nitrocellulose, the filter was hybridized with pHR28, (a human ribosomal), or pAM91 (a mouse actin) DNA probe. As shown in the autoradiographs (Figure 1d and 1e) and confirmed by scanning the dots, there is no substantial difference in the amount of ribosomal or actin RNA present in these samples.

*Northern blot hybridization analysis*

Northern blot hybridization analysis was carried out to measure the sizes of c-*onc* related transcripts. A v-Ki-*ras* probe, HiHi3 recombinant (Ellis *et al.* 1981), revealed the presence of one main band of ~5.8 kb in total cellular RNA (Figure 2a and c). In several cases a much less intense band of ~2.2 kb was seen as well as some other nondiscrete hybridization probably due to degradation or possibly to cross hybridization with other *ras* gene family transcripts. As shown in Figure 2b, d after scanning across this 5.8 kb band, the ki-*ras* related transcript was found in higher amounts in premalignant and malignant tissues of the colorectum as compared to normal mucosa. A similar sized major transcript was also found in RNA from HL60 and K562 cells. As shown in Figure 2e when a v-Ha-*ras* probe, BS9 recombinant (Ellis *et al.*, 1980) was used, the same size band of 5.8 kb was also present, again more intensely in some premalignant and malignant tissues as compared to normal colorectal mucosa (Figure 2f). These results confirm and extend the spot hybridization analyses. The nature of these transcripts was further investigated by isolating polyA$^+$ RNA and Northern blot hybridization analysis. As shown in Figure 2g, i, Ki-*ras* and Ha-*ras* related transcripts of 5.8 kb in size were again found to be elevated in premalignant and malignant tissues as compared to normal colorectal mucosa. We also detected the 5.8 kb sized transcripts using pT24-C3 (Santos *et al.*, 1982), a recombinant carrying a 6.6 kb Bam HI human DNA fragment containing the whole bladder carcinoma oncogene c-Ha-*ras* 1 (data not shown). The exact nature of these transcripts is still incompletely understood. However mechanisms accounting for varied Ki-*ras* transcripts in terms of alternative splicing patterns have been recently described which include the generation of such a 5.8 kb species (Shimizu *et al.*, 1983; McGrath *et al.* 1983). It seems most likely that the same sized transcript seen with the Ha-*ras* probe represents in fact hybridisation with products of another member of the *ras* gene family. Northern blot hybridization analysis of total of polyA$^+$ RNA using a *sis* probe failed, however, to show any discrete RNA transcripts.

### Discussion

Other studies with RNA from human cells have demonstrated the presence of 1.2 kb Ha-*ras* related transcripts in the T24 human bladder carcinoma cell line (Goldfarb *et al.*, 1982) and two ~6.0 kb Ha-*ras* related transcripts in human haematopoietic cell lines (Westin *et al.*, 1982b). More recently, using an N-*ras* probe 3 different sized transcripts of 5.8, 2.2 and 1.5 kb have been found in normal human fibroblasts and established human cell lines and it has been claimed that the 2.2 kb transcript is related to the N-*ras* oncogene (Hall *et al.*, 1983).

The human genome contains at least four genes homologous to the transforming genes of Kirsten

**Figure 2** Northern blot hybridization analysis of transcripts related to human Ki-*ras* and Ha-*ras* oncogenes in RNAs from samples of normal premalignant and malignant tissues of the colorectum. Total RNAs were isolated as described in **Materials and methods.** Poly A$^+$ RNA was isolated using an oligo(dT)-cellulose Type 3 from Collaborative Research Inc. (Spandidos & Paul, 1982). In (a), (c) and (e), 20 $\mu$g of total cell RNA and in (g) and (i) poly A$^+$ RNA isolate from 100 $\mu$g total cell RNA were analyzed in 1% agarose-formaldehyde-containing gels, blotted on to nitrocellulose and hybridized with *onc* probes. The HiHi3 (Ellis *et al.*, 1981) recombinant containing the v-Ki-*ras* sequences was used as a probe in panels (a), (c) and (g). The BS9 (Ellis *et al.*, 1980) recombinant containing the v-Ha-*ras* sequences was used in (e) and (i). N = normal colorectal mucosa, P = premalignant polyps, M = malignant adenocarcinomata. The autoradiographs are shown in (a), (c), (e), (g) and (i) and the scans across the 5.8 kb bands in (b), (d), (f), (h) and (j).

a

Patient no.

Histology kb

5.8

28S

18S

HiHi3 (V-Ki-ras) probe b c

Cell lines

K562

HL60

CHB

Patient no.

Histology

28S

18S

HiHi 3 (V-Ki-ras) probe d e

Cell lines

Patient no.

Histology

HL60

K562

kb

5.8

28S

18S

BS9 (V-Ha-ras) probe f g poly A*

i poly A*

Patient no.

Histology kb

5.8

28S

18S

HiHi3 (V-Ki-ras)

BS9 (V-Ha-ras)

h j and Harvey murine sarcoma viruses (Chang *et al*., 1982) which are dispersed in different chromosomes (O'Brien *et al*., 1983). Moreover, recent transfection studies have revealed the presence of a distantly related N-*ras* oncogene (Hall *et al*., 1983). Our results demonstrate that cellular sequences related to the transforming genes of Kirsten and Harvey murine retroviruses are actively transcribed in human tissues. The demonstration that Ki-*ras* and Ha-*ras* related transcripts are elevated in premalignant and malignant tissues as compared to normal colorectal mucosa shows that the expression of these *onc* genes is associated with the transformed state of the cells and suggests that elevated expression of these genes in premalignant state(s) may be critical in the process of carcinogenesis. The fact, however, that only a relatively small proportion of these premalignant polyps progress to frank malignancy although elevated oncogene expression was observed in all, suggests that, consistent with the concept of carcinogenesis being a multi-step process (Spandidos, 1983) the elevation observed here is not in itself sufficient to produce malignant change. Parallel studies in this laboratory, of carcinogen induced mouse skin papillomata, which have a similar potential for malignancy, have also demonstrated elevated Ha-*ras* oncogene expression and in addition, the DNA from these tumours has acquired transforming activity in transfection assays (Balmain *et al*., 1984).

The cellular homologues of several retroviral oncogenes have been shown to exhibit tissue-specific patterns of transcriptional activity (Westin *et al*., 1982*a*, *b*, Gonda *et al*., 1982). Expression of c-*onc* genes during mouse development (Muller *et al*., 1982; 1983) and liver regeneration (Goyette *et al*., 1983) has lent further support to the hypothesis that cellular oncogenes play a role in normal developmental processes. Abnormal expression of these genes could be directly involved in the development of the transformed phenotype of tumour cells. Although meaningful *in vivo* studies of proliferation rates in these tumours involving repeated sampling and labelling would present obvious ethical problems, the most recent studies on human material *in vitro* comparing malignant tumours to normal mucosa using stathmokinetic (Pritchett *et al*., 1982) and $^{3}$H-Thymidine labelling (Bleiburg *et al*., 1976) techniques suggest that cell birth rate and turnover time respectively, were only very slightly increased in tumours or not significantly different. Similar findings have been reported for premalignant polyps in human (Weisburger *et al*., 1975), and in experimentally induced rodent tumours, where similar proliferation rates for benign and malignant tumours were reported (Sunter *et al*., 1980). In the tumours, furthermore, the growth fraction is probably actually lower than in the normal mucosa. Thus the marked elevation of *ras* related transcripts we observe would not appear to be comparable to the two–three fold increase seen in regenerating liver (Goyette *et al*., 1983).

Gene amplification seems not to be involved in the generation of elevated *onc* transcript levels found in our present study since Ha-*ras* and Ki-*ras* related DNA sequences in the various tissues were at approximately the same level when examined by DNA spot hybridization analysis (data not shown). However, oncogene amplification remains a possibility particularly since we have observed such a phenomenon in DNA from a different adenocarcinoma of the colon (our unpublished results). Such a phenomenon has recently been described for c-*myc* (Collins & Groudine, 1982; Dala-Favera *et al*., 1982) and Ki-*ras* (Schwab *et al*., 1983).

Since our results demonstrate that both the premalignant and malignant tumours examined here are characterized by elevated levels of *ras* family transcripts and if the gene product is unaltered, the question obviously arises as to the nature of the further event(s) involved in the acquisition of the malignant phenotype and their relationship with the changes in gene expression observed here. To address this question may require the use of further assay systems, although as an initial step it will clearly be of interest to clone the *ras* genes involved directly from the tumours since they may not readily be detected in transfection experiments, and such studies, as well as transfection studies using DNA from these tumours, are currently in progress in our laboratory.

We thank members of the Department of Pathology, Glasgow Royal Infirmary, for their co-operation and in particular Professor R.B. Goudie and Dr F.D. Lee for helpful advice, and Messrs. C.S. McArdle, I.G. Finlay and C.S. Morran of the Department of Surgery, Glasgow Royal Infirmary for facilitating the supply of specimens. The pHR28 ribosomal probe was prepared by A. Sproul. We are also indebted to Drs J. Paul, J. Neil, A. Balmain, G.D. Birnie and N.M. Wilkie of the Beatson Institute for helpful discussions. I.B.K. is currently a CRC Research Fellow in the Department of Surgery, Glasgow Royal Infirmary and during the initial stages of this work was McGhie Cancer Research Fellow, Department of Pathology, Glasgow Royal Infirmary. The Beatson Institute is supported by the Cancer Research Campaign of Great Britain.

### References

BALMAIN, A., RAMSDEN, M., BOWDEN, G.T. & SMITH. J. (1984). Activation of the mouse cellular Harvey-*ras* gene in chemically induced benign skin papillomas. *Nature*, **307**, 658.

BLEIBERG, H. & GALAND, P. *In vitro* autoradiographic determination of cell kinetic parameters in adenocarcinomas and adjacent healthy mucosa of the human colon and rectum. *Cancer Res.*, **36**, 325.

CHANG, E.H., GOUDA, M.A., ELLIS, R.W., SCOLNICK, E.M. & LOWY, D.R. (1982). Human genome contains four genes homologous to transforming genes of Harvey and Kirsten murine sarcoma viruses. *Proc. Natl Acad. Sci.*, **79**, 4848.

COLLINS, S. & GROUDINE, M. (1982). Amplification of endogenous myc-related DNA sequences in a human myeloid leukaemia cell line. *Nature*, **298**, 679.

DALLA FAVERA, R., GELMANN, E.P., GALLO, R.C. & WONG-STAAL, F. (1981). A human one gene homologous to the transforming gene (v-sis) of simian sarcoma virus. *Nature*, **292**, 31.

DALLA FAVERA, R., WONG-STAAL, F. & GALLO, R.C. (1982). Onc gene amplification in promyelocytic leukaemia cell line HL60 and primary leukaemic cells of the same patient. *Nature*, **299**, 61.

DENHARDT, D.T. (1966). A membrane filter technique for the detection of complementary DNA. *Biochem. Biophys. Res. Commun.*, **23**, 641.

ELLIS, R.W., DEFEO, D., MARYAK, J.M. & 5 others (1980). Dual evolutionary origin for the rat genetic sequences of Harvey murine sarcoma virus. *J. Virol.* **36**, 408.

ELLIS, R.W., DEFEO, D., SHIH, T.Y. & 5 others (1981). The p21 src genes of Harvey and Kirsten sarcoma viruses originate from diverent members of a family of normal vertebrate genes. *Nature*, **292**, 506.

EVA, A., ROBBINS, K.C., ANDERSEN, P.R. & 11 others. (1982). Cellular genes analogous to retroviral onc genes are transcribed in human tumour cells. *Nature*, **295**, 116.

GOLDFARB, M., SHIMIZU, K., PERUCHO, M. & WIGLER, M. (1982). Isolation and preliminary characterization of a human transforming gene from T24 bladder carcinoma cells. *Nature*, **296**, 404.

GONDA, T.J., SHEINESS, D.K. & BISHOP, J.M. (1982). Transcripts from the cellular homologs of retroviral oncogenes: distribution among chicken tissues. *Mol. Cell. Biol.* **2**, 617.

GOYETTE, M., PETROPOULOS, C.J., SHANK, P.R. & FAUSTO, N. (1983). Expression of a cellular oncogene during liver regeneration. *Science*, **219**, 510.

HALL, A., MARSHALL, C.J., SPURR, N.K. & WEISS, R.A. (1983). Identification of transforming gene in two human sarcoma cell lines as a new member of the *ras* gene family located on chromosome 1. *Nature*, **303**, 396.

JASS, J.R. (1983). Relation between metaplastic polyp and carcinoma of the colorectum. *Lancet*, **i**, 28.

LASKEY, R.A. & MILLS, A.D. (1977). Enhanced autoradiographic detection of $^{32}$ and $^{125}$I using intensifying screens and hypersensitized films. *FEBS Letters*, **82**, 314.

MCCOY, M.S., TOOLE, J.J., CUNNINGHAM, J.M., CHANG, E.H., LOWY, D.R. & WEINBERG, R.A. (1983). Characterization of a human colon/lung carcinoma oncogene. *Nature*, **302**, 79.

MCGRATH, J.P., CAPON, D.J., SMITH, D.H. & 4 others. (1983). Structure and organization of the human Ki-*ras* proto-oncogene and a related processed pseudogene. *Nature*, **304**, 501.

MINTY, A.J., ALONSO, S., CARAVATTI, M. & BUCKINGHAM, M.E. (1982). A fetal skeletal muscle actin mRNA in the mouse and its identity with cardiac actin mRNA. *Cell*, **30**, 185.

MORSON, B.C. (1974). Evolution of cancer of the colon and rectum. *Cancer*, **34**, 845.

MULLER, R., SLAMON, D.J., TREMBLAY, J.M., CLINE, M.J. & VERMA, I.M. (1982). Differential expression of cellular oncogenes during pre- and postnatal development of the mouse. *Nature*, **299**, 640.

MULLER, R., VERMA, I.M. & ADAMSON, E.D. (1983). Expression of c-onc genes: c-fos transcripts accumulate to high levels during development of mouse placenta, yolk sac and amnion. *EMBO J.*, **2**, 679.

O'BRIEN, S.J., NASH, W.G., GOODWIN, J.L., LOWY, D.R. & CHANG, E.H. (1983). Dispersion of the *ras* family of transforming genes to four different chromosomes in man. *Nature*, **302**, 839.

PRITCHETT, C.J., SENIOR, P.V., SUNTER, J.P., WATSON, A.J., APPLETON, D.R. & WILSON, R.G. (1982). Human colorectal tumours in short-term organ culture. A stathmokinetic study. *Cell Tissue Kinet.*, **15**, 555.

PULCIANI, S., SANTOS, E., LAUVER, A.V., LONG, L.K., AARONSON, S.A. & BARBACID, M. (1982). Oncogenes in solid human tumours. *Nature*, **300**, 539.

RIGBY, P.W.J., DIECKMANN, M., RHODES, C. & BERG, P. (1977). Labelling deoxyribonucleic acid to high specific activity in vitro by nick translation with DNA polymerase I. *J. Mol. Biol.* **113**, 237.

ROGNUM, T.O. & BRANDTZAEG, P. (1983). How reliable in terms of oncogenic development are functional markers of colorectal cancer? *Lancet*, **i**, 239.

SANTOS, E., TRONICK, S.R., AARONSON, S.A., PULCIANI, S. & BARBACID, M. (1982). T24 human bladder carcinoma oncogene is an activated form of the normal human homologue of BALB- and Harvey-MSV transforming genes. *Nature*, **298**, 343.

SCHWAB, M., ALITALO, K., VARIMUS, H.E., BISHOP, J.M. & GEORGE, D. (1983). A cellular oncogene (c-Ki-*ras*) is amplified, overexpressed, and located within karyotypic abnormalities in mouse adrenocortical tumour cells. *Nature*, **303**, 497.

SHIMIZU, K., BIRNBAUM, D., RULEY, M.A. & 6 others. (1983). Structure of the Ki-*ras* gene of the human lung carcinoma cell line Calu-1. *Nature*, **304**, 497.

SPANDIDOS, D.A. (1983). Cellular oncogenes mutations and cancer. *Anticancer Res.*, **3**, 121.

SPANDIDOS, D.A. & PAUL, J. (1982). Transfer of human globin genes to erythroleukemic mouse cells. *EMBO. J.*, **1**, 15.

SPANDIDOS, D.A., HARRISON, P.R. & PAUL, J. (1981). Transfer and expression of herpes simplex virus thymidine kinase and human globin genes in mammalian cells studies by spot hybridization. *Biosci. Rep.*, 1911.

SUNTER, J.P., HULL, D.L., APPLETON, D.R. & WATSON, A.J. (1980). Cell proliferation of colonic neoplasms in dimethylhydrazine-treated rats. *Br. J. Cancer*, **42**, 95.

WAHL, G.M., STERN, M. & STARK, G.R. (1979). Efficient transfer of large DNA fragments from agarose gels to diazobenzyloxymethyl-paper and rapid hybridization by using dextran sulphate. *Proc. Natl Acad. Sci.*, **76**, 3683.

WEISBURGER, J.H., REDDY, B.S. & JOFFEY, D.L. (1975). In: *Colo-Rectal Cancer*, UICC Technical Report Series, Vol. 19, no. 2, p. 72.

WESTIN, E.H., GALLO, R.C., ARYA, S.K. & 5 others. (1982*a*). Differential expression of the amv gene in human hematopoietic cells. *Proc. Natl Acad. Sci.*, **79**, 2194.

WESTIN, E.H., WONG-STAAL, F., GELMANN, E.P. & 5 others. (1982*b*). Expression of cellular homologues of retroviral onc genes in human hematopoietic cells. *Proc. Natl Acad. Sci.*, **79**, 2490.